# Supergroup C *Wolbachia*, mutualist symbionts of filarial nematodes, have a distinct genome structure

**DOI:** 10.1098/rsob.150099

**Published:** 2015-12-02

**Authors:** Francesco Comandatore, Richard Cordaux, Claudio Bandi, Mark Blaxter, Alistair Darby, Benjamin L. Makepeace, Matteo Montagna, Davide Sassera

**Affiliations:** 1 Dipartimento di Scienze Veterinarie e Sanità Pubblica (DIVET), Università degli Studi di Milano, Milano, Italy; 2 Dipartimento di Scienze Agrarie e Ambientali, Università degli Studi di Milano, Milano, Italy; 3 Dipartimento di Biologia e Biotecnologie, Università degli Studi di Pavia, Pavia, Italy; 4 Université de Poitiers, UMR CNRS 7267 Ecologie et Biologie des Interactions, Equipe Ecologie Evolution Symbiose, Poitiers, France; 5 Institute of Evolutionary Biology and Centre for Immunity, Infection and Evolution, The School of Biological Sciences, University of Edinburgh, Edinburgh EH9 3TF, UK; 6 Institute of Integrative Biology and the Centre for Genomic Research, University of Liverpool, Liverpool L69 7ZB, UK; 7 Institute of Infection and Global Health, University of Liverpool, Liverpool L3 5RF, UK

**Keywords:** *Wolbachia*, GC skew, filarial nematodes, genome characteristics

## Abstract

*Wolbachia pipientis* is possibly the most widespread endosymbiont of arthropods and nematodes. While all *Wolbachia* strains have historically been defined as a single species, 16 monophyletic clusters of diversity (called supergroups) have been described. Different supergroups have distinct host ranges and symbiotic relationships, ranging from mutualism to reproductive manipulation. In filarial nematodes, which include parasites responsible for major diseases of humans (such as *Onchocerca volvulus*, agent of river blindness) and companion animals (*Dirofilaria immitis,* the dog heartworm), *Wolbachia* has an obligate mutualist role and is the target of new treatment regimens. Here, we compare the genomes of eight *Wolbachia* strains, spanning the diversity of the major supergroups (A–F), analysing synteny, transposable element content, GC skew and gene loss or gain. We detected genomic features that differ between *Wolbachia* supergroups, most notably in the C and D clades from filarial nematodes. In particular, strains from supergroup C (symbionts of *O. volvulus* and *D. immitis*) present a pattern of GC skew, conserved synteny and lack of transposable elements, unique in the *Wolbachia* genus. These features could be the consequence of a distinct symbiotic relationship between C *Wolbachia* strains and their hosts, highlighting underappreciated differences between the mutualistic supergroups found within filarial nematodes.

## 1. Background

*Wolbachia* is one of the most widespread and studied genera of intracellular bacteria, encompassing endosymbionts of arthropods and nematodes [1,2]. All *Wolbachia* strains have historically been classified into a single species, *Wolbachia pipientis* [3,4]. This species, however, on the basis of single gene and multi-locus phylogenies [5,6], has been divided into 16 monophyletic supergroups, labelled A–Q (as supergroup G is possibly an artefacts we have not included it in the total of 16 considered here) [4,7,8]. The (A,B),(D,(C,F)) phylogenetic relationship among the most studied supergroups has recently been confirmed using whole-genome phylogenetic approaches, albeit only on a limited number of strains [9–11]. The taxonomic status of the major *Wolbachia* lineages is contentious [4,12]. While a ranking to species level has recently been proposed [13,14] based on genome analyses, this pivotal change in *Wolbachia* classification does not include all current supergroups and remains to be accepted by the *Wolbachia* community. Thus, in this work, we have used the historical *Wolbachia* nomenclature (one species, 16 supergroups).

The different *Wolbachia* supergroups are associated with distinct sets of hosts in arthropoda and nematoda. The nature of the association between *Wolbachia* strains and their hosts also varies greatly. The symbiosis between C and D supergroup strains and their filarial nematode hosts presents features associated with mutualism, including 100% prevalence [15], strict vertical inheritance [1,16] and metabolic integration [17–19]. Because filarial nematodes are responsible for major neglected tropical diseases of humans (including onchocerciasis or river blindness, caused by *Onchocerca volvulus*, and lymphatic filariasis, caused by *Brugia malayi* among other species), alongside an important infection of companion animals (heartworm, caused by *Dirofilaria immitis*), this obligate relationship has been exploited for novel anti-filarial treatments, such that the nematodes are sterilized or killed by antibiotics [20–22]. In contrast, A and B supergroup strains, infecting arthropod hosts, have less than 100% prevalence, display evidence of rampant lateral transfer and induce a variety of reproductive manipulation phenotypes, including cytoplasmic incompatibility, parthenogenesis, killing of male embryos and feminization of genetic males [2,23]. *Wolbachia* strains of the F supergroup have been observed in association with both arthropods and nematodes [4,24].

A recent genomic study, focused on two strains of *Wolbachia* belonging to either supergroup A or B and co-infecting *Drosophila simulans*, showed a lack of genetic exchange, suggesting their genetic isolation [14]. Are these results by Ellegaard *et al.* unique within the genus, or is genetic isolation common among *Wolbachia* lineages? If the different supergroups experienced independent evolution, then we can expect their genomes to present specific features as a consequence of their independent evolutionary histories.

*Wolbachia* strains have reduced genome size, a feature observed in most endosymbiont bacteria [25–27]. The process of genome reduction in endosymbionts can be classified in four stages [28], as follows. (i) Free-living bacteria: large genome size, few transposable elements, gene acquisition and loss, interstrain recombinations. (ii) Recently host-restricted bacteria: genome size smaller than free-living bacteria, many transposable elements, chromosome rearrangements and loss of genomic regions. (iii) Long-term obligate symbionts: further reduced genome size, stable chromosome and few or no transposable elements. (iv) Tiny-genome symbionts: very small genome size and high chromosome stability.

In this work, we compared the genomes of *Wolbachia* strains belonging to the A–D and F supergroups, in order to identify conserved and variable genomic features. We considered intragenomic recombinations, transposable elements, chromosome rearrangements, mutational bias and gene loss or gain. We found that *Wolbachia* strains belonging to supergroup C have conserved and distinct genomic features, probably the result of extensive periods of independent evolution.

## 2. Methods

### 2.1. Dataset

The genome assemblies of eight *Wolbachia* strains belonging to A–D and F supergroups (*w*Mel, *w*Ri, *w*PipPel, *w*Di, *w*Oo, *w*Bm, *w*Ls and *w*Cle) and of seven other Alphaproteobacteria (*Caulobacter crescentus* strain CB15, Cre; *Anaplasma centrale* strain Israel*,* Ace; *Anaplasma phagocytophilum* strain HZ, Aph; *Ehrlichia chaffeensis* strain Arkansas, Ech; *Ehrlichia ruminantium* strain Gardel, Eru; *Neorickettsia risticii* strain Illinois, Nri; *Neorickettsia sennetsu* strain Miyayama, Nse) were retrieved from public database (for more information about genome features, see table 1). *Caulobacter crescentus* was chosen because it is a complete genome of an alphaproteobacterium for which origin and terminus of replication were experimentally determined [29]. The genome assemblies included in the study are all complete or almost complete, with the exception of the genome of *w*Ls, which is divided into 10 contigs. We included the genome of *w*Ls in the study as a second representative of the nematode-associated *Wolbachia* supergroup D.

**Table 1.** List of the genomes included in this study. For each genome, information about the strain, the corresponding host and the genome are reported.

*Wolbachia* strains (short name)	hosts	supergroups	no. contigs	contig length (nt)	sources
*w*Mel	*Drosophila melanogaster*	A	1	1 267 782	NC_002978
*w*Ri	*Drosophila simulans*	A	1	1 445 873	NC_012416
*w*PipPel	*Culex quinquefasciatus*	B	1	1 482 455	NC_010981
*w*Oo	*Onchocerca ochengi*	C	1	957 990	HE660029
*w*Di	*Dirofilaria immitis*	C	2	919 954, 1058	http://dirofilaria.nematod.es
*w*Bm	*Brugia malayi*	D	1	1 080 084	NC_006833
*w*Ls	*Litomosoides sigmodontis*	D	10	605 213, 245 144, 135 750, 38 729, 16 626, 5094, 1163, 500, 375, 342	http://litomosoides.nematod.es
*w*Cle	*Cimex lectularius*	F	1	125 0060	AP013028

Outgroup strains (short name)	strain names	supergroups	no. contigs	contig length (nt)	sources
Ace	*Anaplasma centrale* str. Israel	—	1	1 206 806	NC_013532
Aph	*Anaplasma phagocytophilum* HZ	—	1	1 471 282	NC_007797
Ech	*Ehrlichia chaffeensis* str. Arkansas	—	1	1 176 248	NC_007799
Eru	*Ehrlichia ruminantium* str. Gardel	—	1	1 499 920	NC_006831
Nri	*Neorickettsia risticii* str. Illinois	—	1	879 977	NC_013009
Nse	*Neorickettsia sennetsu* str. Miyayama	—	1	859 006	NC_007798
Ccr	*Caulobacter crescentus* CB15	—	1	4 016 947	NC_002696

### 2.2. Origin of replication and genome orientation

The genomes of the *Wolbachia* strains included in the study were aligned with progressiveMauve [30]. For each genome, the position of the origin of replication (ORI) was inferred on the basis of the *w*Mel and *w*Bm ORI positions proposed by Ioannidis *et al.* [31]. Each genome assembly was oriented following the *w*Mel and *w*Bm ORI orientation, and organized to start with the ORI position. Below, we refer to these reorganized genomes as ‘ORI-starting’ genomes.

### 2.3. Analysis of genome rearrangements

Pairwise genome alignments of the *w*Mel, *w*Ri, *w*PipPel, *w*Di, *w*Oo, *w*Bm, *w*Ls and *w*Cle *Wolbachia* strains were produced and plotted with the software MUMmer v. 3.0 [32].

### 2.4. Transposable elements

Insertion sequences (ISs) and group II introns were identified and annotated in *w*Di (C supergroup), *w*Ls (D supergroup) and *w*Cle (F supergroup). Group II introns were identified following the methods of Leclercq *et al.* [33]. IS elements were identified using ISSaga [34], followed by manual curation of ISSaga output files. For *w*Ls, most ISSaga hits were short and often formed groups of two to four hits located next to each other. This is typical of pseudo-genized and degraded IS elements. We attributed two consecutive hits to the same or to distinct IS copies using the following rules:

(1) IS family: if the two hits belong to different IS families, then they belong to distinct copies. Otherwise, go to criterion (2).
(2) Orientation: if the two hits are in opposite orientation, then they belong to distinct copies. Otherwise, go to criterion (3).
(3) Physical distance: if distance between the two hits is greater than 300 bp, then they belong to distinct copies. Otherwise, they belong to the same copy.

### 2.5. GC skew

The cumulative GC skew curve was calculated for each of the ORI-starting *Wolbachia* genome assemblies. It was calculated applying the formula *Σ*G − C/G + C, with a window size of 1000 nt and step size of 100 nt (analyses were performed with an in-house Perl script).

For each of the *Wolbachia* strains in the dataset, with the exception of *w*Ls (fragmented in 10 contigs), the *potential effect* of genomic rearrangements on the current GC skew curve was evaluated. The following procedure was used: (i) the ORI-starting genome was aligned against the ORI-starting *w*Di genome with progressiveMauve; (ii) the detected syntenic blocks were sorted and oriented according to the ORI-starting *w*Di order; (iii) the cumulative GC skew curves were calculated for both the obtained reoriented genome and relative aligned *w*Di genome; and (iv) the mean absolute difference between the two curves was calculated. The mean distance values calculated for all *Wolbachia* strains were compared with the Wilcoxon–Mann–Whitney test with Bonferroni *post hoc* correction.

### 2.6. Mutational bias

The effect of mutational bias on the guanine and cytosine distribution along the genomes of *Wolbachia* strains C and F (*w*Di, *w*Oo—C supergroup; *w*Cle—F supergroup) was evaluated using *Wolbachia* strains A, B and D (*w*Mel, *w*Ri—A supergroup; *w*PipPel—B supergroup; *w*Ls and *w*Bm—D supergroup) as outgroups. A dataset of single-copy orthologous genes, shared among all the eight *Wolbachia* strains included in the study, was obtained with OrthoMCL [35] and in-house Perl scripts. Nucleotide gene sequences were aligned on the corresponding amino acid alignments, using Muscle [36] and in-house Perl scripts. For each gene, the number of mutations towards G and towards C for third position residues was evaluated for each pair of *Wolbachia* strains, using a custom Perl script. The mutational biases along *w*Di, *w*Oo and *w*Cle genomes were evaluated comparing each of them against all the other seven *Wolbachia* strains included in the study. The mutational biases on the Watson (forward) and Crick (reverse) strands (*sensu lato*) were evaluated by calculating the respective bias indexes. For genes located on the Watson strand, the bias index was computed as the ratio between the number of mutations towards G and the number of mutations towards C. Conversely, for genes located on the Crick strand, the bias index was computed as the ratio of the number of mutations towards C and the number of mutations towards G. The average of the middle positions of the genes with bias index more than one and less than one were compared with the Wilcoxon–Mann–Whitney test.

### 2.7. Gene loss and gain

Events of gene loss/gain that occurred in the genome of the ancestor of *Wolbachia* supergroup C were inferred on the basis of the pattern of gene presence/absence in the present strains. This presence/absence pattern was reconstructed, annotating the genomes of the eight *Wolbachia* strains included in the study and of six *Anaplasmataceae* outgroups, against the clusters of orthologous groups (COGs) database by PSI-BLAST with a *p*-value cut-off of 10^–5^. The loss and gain events occurred in the genome of the ancestor of *Wolbachia* supergroup C were inferred using the Gloome tool [37], mapping the pattern of presence/absence of functional COG annotations on a phylogenetic tree reconstructed from the literature [9–11,38]. The Gloome tool confers a probability value to each inferred event. Only events with a probability greater than 75% were considered reliable and thus manually checked.

## 3. Results

We are interested in the evolutionary dynamics of *Wolbachia*, an important genus of intracellular bacteria. Here, we explore the genomic signatures in eight *Wolbachia* strains from supergroups A to F, including intragenomic recombination, transposable elements, GC skew curve, mutational bias and gene loss or gain. We focus specifically on differences between two supergroup C genomes, *w*Di (from the dog heartworm, *D. immitis*) and *w*Oo (from *Onchocerca ochengi*, a bovine parasite very closely related to *O. volvulus*); and two supergroup D genomes, *w*Bm (from a human lymphatic filariasis parasite, *B. malayi*) and *w*Ls (from a filarial model of rodents, *Litomosoides sigmodontis*).

### 3.1. Intragenomic recombinations

**Figure 1.**
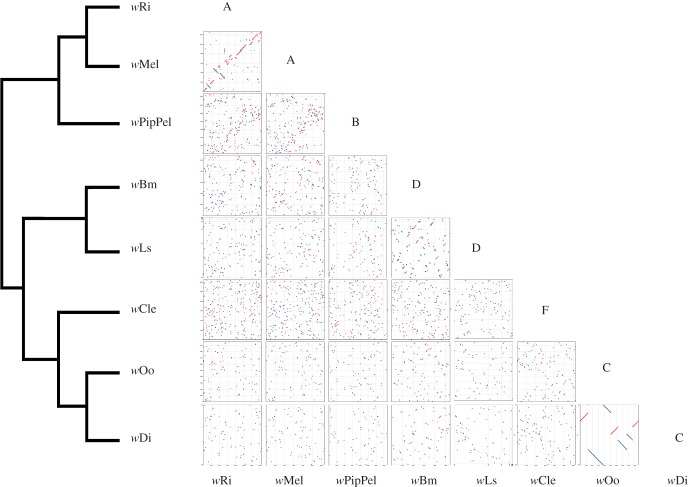
Synteny conservation in supergroup C *Wolbachia.* A graphic representation of MUMmer v. 3.0 output is shown in the dot plots on the right. Red lines display collinear regions, whereas blue lines display inversions. Phylogenetic relationships among the *Wolbachia* strains are shown on the left.

*Wolbachia* genomes have been reported to have undergone extensive rearrangement in comparison with other *Rickettsiales* [39]. We analysed eight genome assemblies belonging to *Wolbachia* strains from supergroups A to F [9,18,40–42]. An alignment of these high-quality genomic assemblies revealed conservation of synteny among the supergroup C genomes *w*Di and *w*Oo, in marked contrast with very low levels of synteny within and between the other supergroups (figure 1). However, the *w*Mel and *w*Ri genomes also show conserved synteny, probably a consequence of their low evolutionary distance [9,42].

### 3.2. Transposable elements

**Figure 2.**
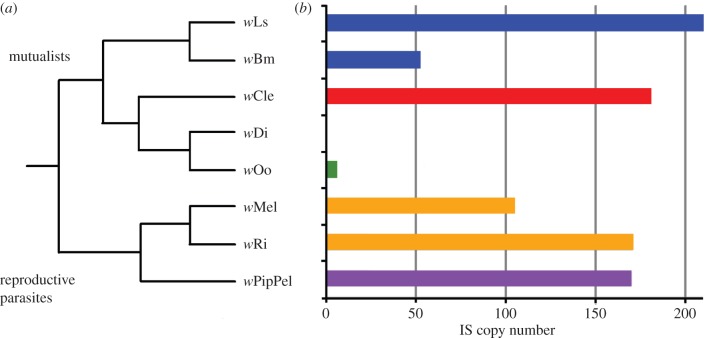
Insertion sequences in *Wolbachia* genomes. (*a*) The known phylogenetic relationships among the *Wolbachia* strains are shown. (*b*) Results of insertion sequence (IS) analyses performed on the *w*Ls, *w*Bm, *w*Di, *w*Oo, *w*Cle, *w*Mel, *w*Ri and *w*PipPel *Wolbachia* strains are displayed as a histogram showing IS quantification. The known phylogenetic relationships among the *Wolbachia* strains are shown in (*a*). For each strain, the corresponding supergroup is colour-coded: orange, A; violet, B; green, C; blue, D; black, E and red, F.

Synteny breakage and recombination is often associated with repeats and transposable elements. We therefore screened the *Wolbachia* genomes for classes of transposable element (electronic supplementary material, table S1; figure 2). We found no group II introns in the *w*Di (C supergroup) and *w*Ls (D supergroup) genomes. However, ISs had a striking, disjointed pattern of presence. While *w*Di had only a single IS (similar to ISWpi16), *w*Ls contained 210 IS copies. Supergroup A and B arthropod *Wolbachia* genomes also have many IS elements [43], albeit fewer than *w*Ls. IS elements cover nearly 12% of the *w*Ls genome, a higher percentage than in any other *Wolbachia* genome sequenced to date. Despite their high copy number, all *w*Ls IS copies appear to be degraded and there is no apparent ‘live’ transpositional activity. Remarkably, 97% of the *w*Ls IS copies (204/210) belong to a single IS type (ISWpi10). The six remaining copies belong to ISWpi5 (electronic supplementary material, table S2). Interestingly, the genome of *w*Cle (F supergroup) is characterized by a high density (10%) and diversity (11 different types) of IS elements and the presence of group II introns (electronic supplementary material, table S1).

Comparing the D supergroup genomes, no IS copy was found to be inserted at an orthologous site, despite the high number of IS copies. By contrast, in supergroup C, the single IS copy found in *w*Di is orthologous to the ISWpi16 copy found in *w*Oo.

### 3.3. GC skew and mutational bias

**Figure 3.**
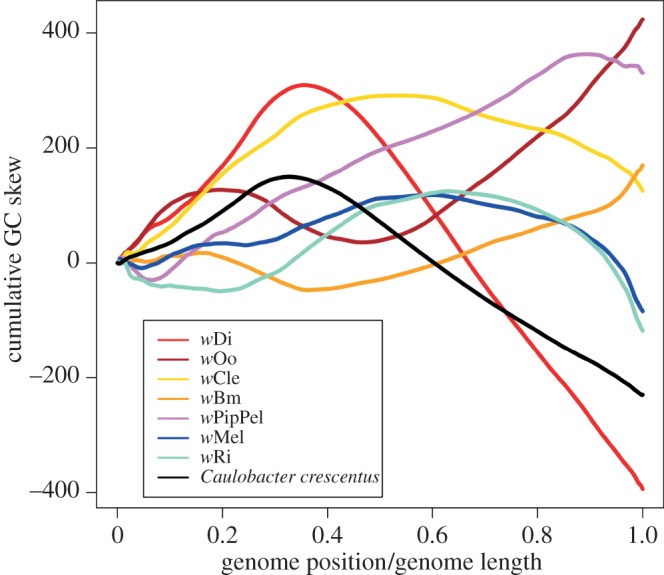
Cumulative GC skew curves. GC skew was calculated with window size of 1000 nucleotides and step size of 100 nucleotides. The curve for *Caulobacter crescentus* is coloured in black, whereas the curves for *Wolbachia* strains are coloured as follows: *w*Mel, blue; *w*Ri, azure; *w*PipPel, pink; *w*Di, red, *w*Oo, dark red; *w*Bm, orange; *w*Cle, yellow.

Another feature described as characteristic of arthropod *Wolbachia* genomes is the absence of strong GC skew [39], in contrast with the pattern commonly observed in most free-living bacteria and in endosymbiotic bacteria such as *Buchnera aphidicola* [44,45]. The cumulative GC skew curve of the seven completely sequenced *Wolbachia* genomes included in the study (*w*Mel, *w*Ri, *w*PipPel, *w*Di, *w*Oo, *w*Bm and *w*Cle) and of the Alphaproteobacterium outgroup, *C. crescentus*, were calculated (figure 2)*.* In agreement with previous analyses on a smaller dataset [39], most *Wolbachia* genomes do not present any genome-wide pattern of GC skew (figure 3). However, the *w*Di genome has a strong pattern of GC skew (figure 3), which, among endosymbionts, is typically observed in bacteria with extremely reduced genomes.

**Figure 4.**
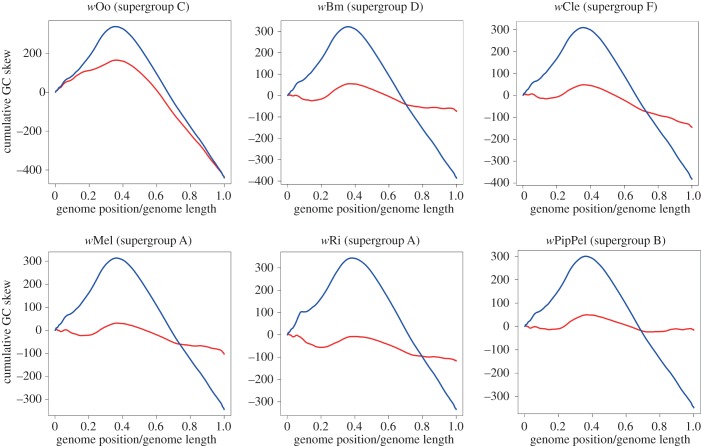
Cumulative GC skew curves of six reoriented *Wolbachia* genomes (red) compared with the *w*Di genome (blue). Genomes were reordered on the basis of the *w*Di gene order using a progressiveMauve genome alignment.

This pattern of cumulative GC skew in *w*Di could have originated uniquely in *w*Di or could be an ancestral feature of *Wolbachia*, lost by most lineages. To test the hypothesis that the *w*Di GC skew pattern is ancestral, we evaluated whether its absence in the other six complete *Wolbachia* genomes included in the study could have been caused by genome rearrangements. We reordered each genome to conform the *w*Di gene order and recalculated the GC skew on the ‘pseudo-ancestral’ genome (figure 4). While rearrangement of supergroup A–C and F genomes did not reveal any hidden GC skew pattern, in the rearranged *w*Oo genome (belonging to the C supergroup), we observed a trend similar to that of *w*Di (figure 4). No better fit was observed between native *w*Di and the other five rearranged *Wolbachia* genomes included in the analysis (*w*Mel, *w*Ri, *w*PipPel, *w*Bm and *w*Cle; electronic supplementary material, figure S1).

**Figure 5.**
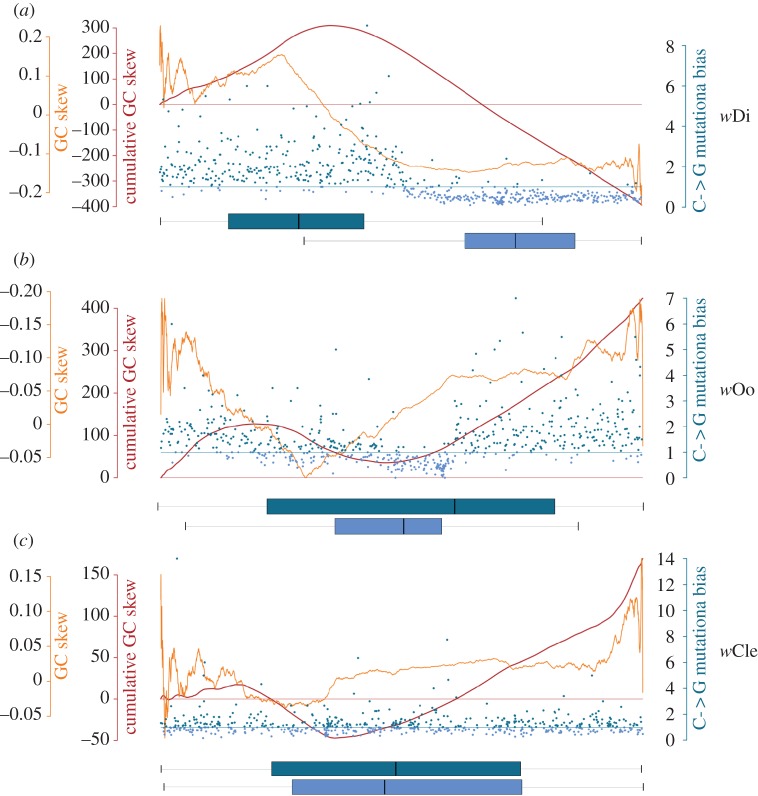
GC mutational bias. The figure displays information about the (*a*) *w*Di, (*b*) *w*Oo and (*c*) *w*Cle genomes, including GC skew, cumulative GC skew and mutational bias calculated using the *w*Bm genome as reference (see Material and methods). In each graph, the position along the genome is reported on the *x*-axis; the GC skew curve is reported with an orange line and the cumulative GC skew with a red line. Genes shared among all the *Wolbachia* strains included in the study are represented with blue/azure coloured points: blue genes have a positive GC mutational bias index, whereas azure genes have a negative GC mutational bias index (see Materials and methods). The horizontal boxplots indicate the average position of genes with positive GC mutational bias index (blue) and with negative GC mutational bias index (azure).

Based on the GC skew analysis presented above, the occurrence of genome rearrangements could explain the difference in GC distribution between *w*Di and the other C supergroup *Wolbachia* genome included in the study (i.e. *w*Oo), but cannot explain the differences between *w*Di and the genomes of strains belonging to other supergroups. We thus hypothesized that, during the evolution of the C supergroup, a mutational bias led to the asymmetric distribution of GC observed in the *w*Di genome. Indeed, in the *w*Di genome, the Watson strand of the genes localized on the first part of the genome tends to be mutated towards G more than towards C, opposite to what was detected in the genes localized on the second part of the genome, as shown in figure 5.

GC skew is thought to arise from biased substitution processes driven by the replicational structure of the circular chromosome. This model explains the opposite mutational biases observed in the genes in the first and in the second part of the *w*Di genome (figure 5*a*). Following this model, we infer that the position of the mutational bias switch, near the middle of the *w*Di genome (figure 5*a*), corresponds to the position of the terminus of replication, but this should be verified experimentally. No mutational bias was observed for the other analysed strains, *w*Oo (supergroup C) and *w*Cle (supergroup F; figure 5*b,c*).

### 3.4. Gene loss and gain in the C *Wolbachia* ancestor

*Wolbachia* genomes vary in size from approximately 0.9 to approximately 1.4 Mb. These size differences could have arisen from either gain of genetic material (including transposable elements and phages) or loss, or both. Gene loss and gain have a strong impact on *Wolbachia* strains’ metabolic capability. Indeed, the genome stability observed in C *Wolbachia* strains, in particular in the *w*Di strain, could be the consequence of specific events of gene loss occurring during the evolution of *Wolbachia* supergroup C.

**Figure 6.**
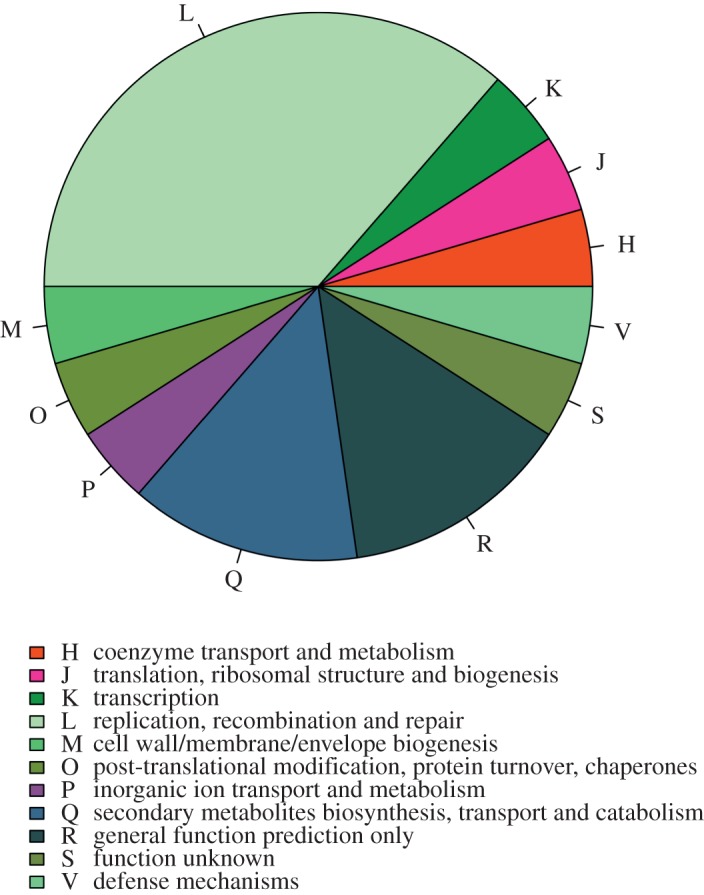
COG classification of the genes identified as putatively lost during the evolution of the *w*Di and *w*Oo ancestor.

We identified the putative events of gene loss and gain in the ancestor of the *Wolbachia* supergroup C, on the basis of the COG annotation of the genomes of the 14 *Anaplasmataceae* strains included in the study (of which eight belong to *Wolbachia*, two to *Anaplasma*, two to *Ehrlichia* and two to *Neorickettsia*). Mapping this COG presence/absence pattern on the *Anaplasmataceae* tree, 22 loss events and no gain events were inferred at node of the C *Wolbachia* strain ancestor (figure 6; electronic supplementary material, table S2). The replication, recombination and repair pathway was affected by a particularly intense erosion process, from which the C *Wolbachia* ancestor lost eight members (figure 6; electronic supplementary maerial, table S3).

## 4. Discussion

Bacteria belonging to the alphaproteobacterial genus *Wolbachia* have been classified into 16 supergroups, mainly on the basis of 16S rDNA phylogenetic analyses. This classification groups *Wolbachia* strains coherently with the host taxonomy and ecology. Phylogenomic analyses have further organized most of the *Wolbachia* diversity into two monophyletic clusters of supergroups: (A + B) and (C + D + F) [9–11]. While recombination has been observed between strains belonging to the same supergroup, each supergroup may be relatively genetically isolated. Indeed, no recombination was detected between *w*Ha (supergroup A) and *w*No (supergroup B), despite their coinfection of the same arthropod species [14]. We can expect that *Wolbachia* strains belonging to a genetically isolated supergroup should present conserved genomic signatures, as a consequence of their independent evolutionary patterns. We sought to detect structural genomic differences between supergroups, with a particular focus on the (C + D + F) cluster.

Early comparisons of *Wolbachia* genomes revealed an extreme lack of synteny between strains from supergroups A and B, and *w*Bm (supergroup D) [39]. Several additional *Wolbachia* genomes belonging to supergroups C, D and F are now available: specifically *w*Di and *w*Oo (supergroup C), *w*Ls (supergroup D) and *w*Cle (supergroup F). This has allowed us to further investigate synteny patterns in the (C + D + F) cluster. Here, we find that the genomes of supergroup C show an elevated level of synteny, compared with the supergroup D genomes included in the study (figure 1). This disjointed pattern suggests that supergroup D genomes may be evolving differently from those of the strains of supergroup C. Similar results, on a slightly different genome dataset, where recently obtained by Ramírez-Puebla *et al*. [13].

IS elements are present in extremely variable numbers in different bacterial lineages, and are known to promote intragenomic recombination, causing the interruption of synteny conservation [46]. *Wolbachia* genomes vary dramatically in terms of their IS content. Supergroup C genomes show a paucity of IS elements, whereas genomes of supergroups A, B, D and F have many IS elements, a pattern consistent with a possible role for IS in synteny breakage in some *Wolbachia* genomes. The low number of IS elements observed in the C *Wolbachia* genomes (ranging from one to six—see electronic supplementary material, table S1) is consistent with the amounts observed in genomes of other long-term, vertically inherited obligate symbionts [28]. Conversely, the genomes of arthropod *Wolbachia* strains included in the study (strains from supergroups A, B and F) contain a higher number of IS elements (ranging from 105 to 181—see electronic supplementary material, table S1), many of which are potentially capable of transposition. This is typical of endosymbionts that undergo at least some horizontal transmission [47]. Interestingly, supergroup D genomes (*w*Bm and *w*Ls) contain a high number of IS elements (respectively 52 and 210—see electronic supplementary material, table S1), but they are all disrupted and on their way to being lost, as part of the reductive genome evolution of these vertically inherited endosymbionts [28]. This is consistent with a scenario in which IS transpositional activity ceased a long time ago in these *Wolbachia* strains, as previously noted for other endosymbionts with a similar lifestyle [28].

In general, lifestyle is thought to be a major factor influencing mobile DNA evolution in intracellular bacteria [47,48]. In *Wolbachia*, the mutualistic supergroup C and D strains are only vertically inherited in their nematode hosts, whereas supergroup A and B strains experience a combination of vertical and horizontal transmission. Horizontal transmission should enable more frequent contact and genetic exchanges with other microorganisms, thereby maintaining a flux of intact IS copies and generating higher IS diversity. The supergroup F genome (from *w*Cle) is also from a strain exhibiting mutualistic interactions with its host, but *w*Cle displays high IS diversity, like the non-mutualistic supergroup A and B strains. This suggests that *w*Cle might have recently shifted to mutualism and still shows transposable element patterns of its non-mutualistic ancestor.

Intragenomic recombinations can affect the distribution of guanine and cytosine along bacterial genomes. Studies on free-living bacterial genomes showed that in many cases, during genome replication, the Watson and Crick strands are subjected to asymmetric cumulative mutation pressures [49,50]. Indeed, intragenomic recombinations randomize the cumulative effect of this mutation pressure. For this reason, the strong asymmetry distribution of cytosine and guanine observed in the *w*Di genome (figure 3) suggests that it experienced a long period of chromosome stability, in contrast with other *Wolbachia* genomes. We reordered the other *Wolbachia* genomes and compared them with *w*Di to identify any residual ancestral GC skew signatures that had not yet been erased during subsequent evolution. The reoriented *w*Oo genome showed stronger GC skew than the natively ordered genome, albeit less pronounced than that of *w*Di, and was more similar to the *w*Di curve than that of other reoriented *Wolbachia* genomes (figure 4).

The analysis of mutational bias on the Watson strand of the *w*Di genome shows that on the genes localized in the first part of the *w*Di genome, mutations towards G are positively selected in comparison with mutations towards C, whereas an inverse pattern is seen in the genes localized on the second part of the *w*Di genome (figure 5). The combination between high genome stability and GC mutational bias probably led to the current asymmetrical distribution of GC along the *w*Di genome. Interestingly, just a weak GC mutational bias can be observed in the *w*Oo genome (figure 5), which currently maintains the GC distribution originated during the evolution of the *w*Oo-*w*Di ancestor. This result suggests that the *w*Oo genome replicates with a very low mutation rate: not enough to generate significant mutational bias, but also not enough to erase the ancestral GC distribution signal conserved in the *w*Di genome.

Klasson & Andersson [45] described an asymmetric distribution of G and C in the genome of the aphid endosymbiont *B. aphidicola*, and hypothesized that the lack of *recA* and mutational bias could be the causes of this GC distribution pattern. Indeed, intragenomic recombination can lead to bacterial death, in the absence of an adequate homologous recombination pathway. *recA,* one of the most important genes involved in the homologous recombination pathway, is lacking in all supergroup C genomes [18,51]. By contrast, in supergroup D, the homologous recombination pathway is complete in the only closed genome available, *w*Bm [40,51], supporting the hypothesis of higher genome plasticity. However, *w*Bm may be exceptional, as other supergroup D genomes appear to have a deficient homologous recombination pathway [51]. It must be noted that these genomes are not closed, thus additional complete genome sequences from supergroup D strains are needed to determine whether *w*Bm is unusual in its *recA* status and rearrangement history.

Is the *w*Di genome representative of the ancestor of all *Wolbachia*? We suggest not. It is likely that the loss of the *recA* pathway in the last common ancestor of supergroup C and the general loss of IS elements resulted in a halt to genome rearrangement, and this stability then permitted a build-up of GC skew and mutational bias in the stabilized genome. Limited subsequent rearrangements observed in *w*Oo have obscured, but not erased, the signatures of evolutionary stability.

The process of gene loss is one of the most important phenomena in the evolution of intracellular bacteria [52]. Within the *Wolbachia* genus, this process is exacerbated in filarial strains, where gene acquisition from other bacterial species has not been described. In our analysis, *recA* was identified as being lost from supergroup C, as expected, but we also identified a number of other losses in the supergroup C lineage associated with a variety of other processes. The physiological linkage between these gene losses, if any, is unclear.

## 5. Conclusion

In conclusion, our analyses present evidence supporting the hypothesis that *Wolbachia* supergroups are not just phylogenetic lineages. Evidence of genetic isolation and convergent evolution had been reported for two strains belonging to *Wolbachia* supergroups A and B [14]. Here, we report evidence that supergroup C strains share a suite of genomic features (very low number of genomic rearrangements, paucity of IS elements, strong GC asymmetric distribution) that is commonly observed in endosymbiotic bacteria with extremely reduced genomes, which have long-lasting relationships with their host. These features are absent in the other lineages of *Wolbachia* included in the study. Genomic analyses enabled us to infer the evolutionary pathway that originated this suite of features. Our results are not sufficient to conclude if the different genomic features observed in C and D supergroup genomes are the result of different selective pressures, or if the two supergroups are in two different stages of the genome reduction process typical of bacterial endosymbionts. Additional genomes will help to shed light on this matter.

Nematode *Wolbachia* strains live in mutualistic association with the host, and are considered important targets for anti-filarial pharmaceutical treatments [41]. In this work, we report genomic evidence that C and D *Wolbachia* supergroup strains experienced a long period of independent evolution. We can hypothesize that the observed differences between the C and D *Wolbachia* strain genomes are a consequence of different specific symbiotic relationships with the filarial hosts, probably resulting in specific host–*Wolbachia* metabolic complementarities. If our results are supported by analyses of additional *Wolbachia* genomes, the mutualism of C and D *Wolbachia* strains with filarial nematodes should be considered separately, with potential implications for anti-*Wolbachia* strategies, as drugs effective against one supergroup may not always be equally potent against the other.

## Supplementary Material

Table S1

## Supplementary Material

Table S2

## Supplementary Material

Table S3

## Supplementary Material

Figure S1
