# Supplementary figures and images for: Supergroup C Wolbachia, mutualist symbionts of filarial nematodes, have a distinct genome structure
Source: Open Biol. 2015 Dec 2;5(12):150099. doi: 10.1098/rsob.150099 (PMC4703054; doi:10.1098/rsob.150099)

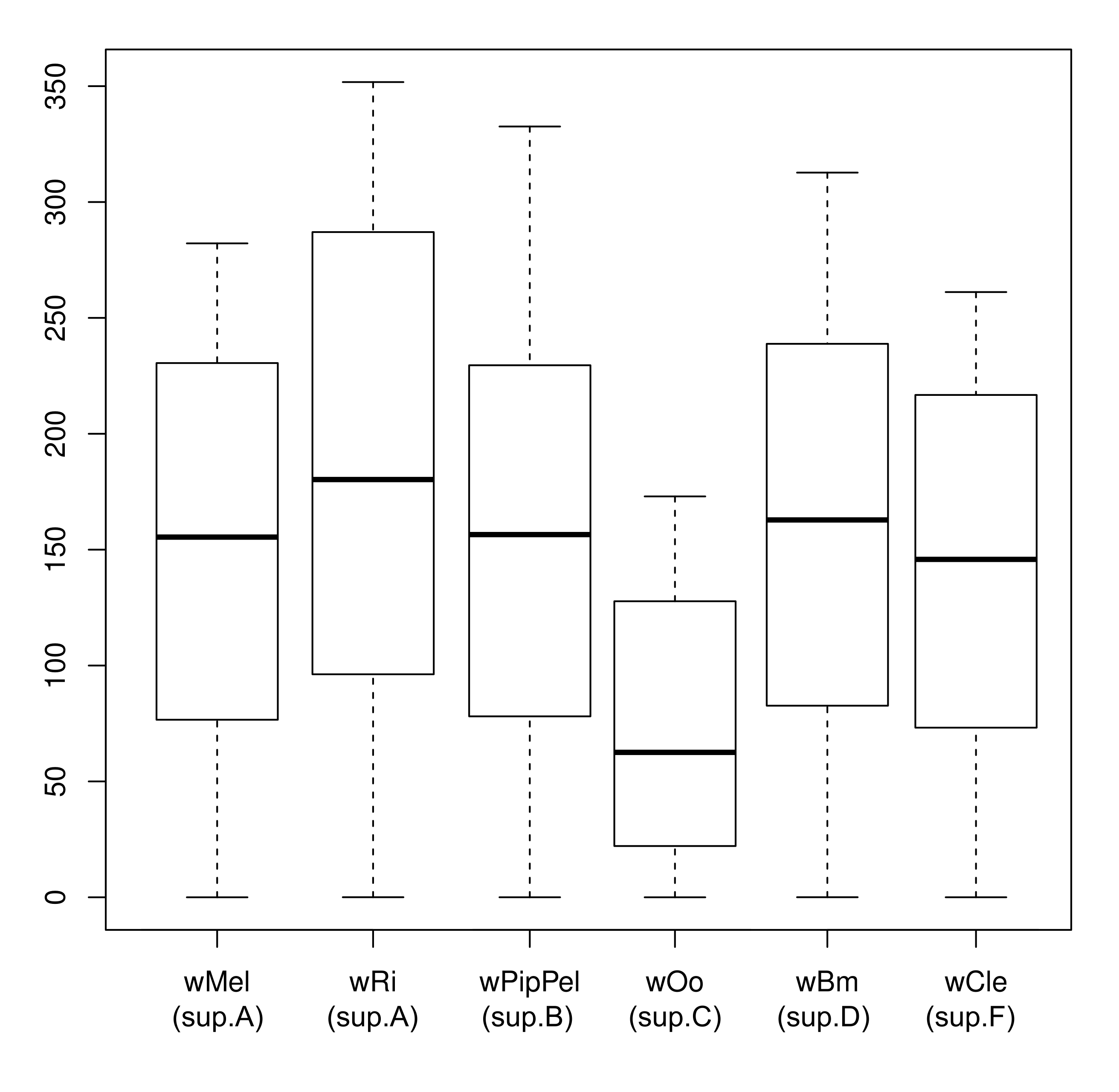

Supplement: Figure S1 [file rsob150099supp4.tif]
